# Supplementary material for: Characteristics and outcomes of hemodialysis patients with COVID-19: a retrospective single center study
Source: PeerJ. 2020 Nov 26;8:e10459. doi: 10.7717/peerj.10459 (PMC7700734; doi:10.7717/peerj.10459)
Supplement: Supplemental Information 1 [file peerj-08-10459-s001.docx]

**Table S1 Laboratory results of 16 hemodialysis patients with COVID-19**

| **Characteristics** | **Hemodialysis Patients with COVID-19 (n = 16)** | | | | | | | | | | | | | | | |  |
| --- | --- | --- | --- | --- | --- | --- | --- | --- | --- | --- | --- | --- | --- | --- | --- | --- | --- |
|  | **Patient 1** | **Patient 2** | **Patient 3** | **Patient 4** | **Patient 5** | **Patient 6** | **Patient 7** | **Patient 8** | **Patient 9** | **Patient 10** | **Patient 11** | **Patient 12** | **Patient 13** | **Patient 14** | **Patient 15** | **Patient 16** | **n (%)** |
| **Admission radiologic findings**  **（chest CT）** |  |  |  |  |  |  |  |  |  |  |  |  |  |  |  |  |  |
| Bilateral patchy shadowing | Yes | Yes | Yes | Yes | Yes | Yes | Yes | Yes | Yes | Yes | Yes | Yes | Yes | Yes | Yes | Yes | 16(100) |
| Ground-glass opacities | Yes | Yes | Yes | Yes | Yes | Yes | Yes | Yes | Yes | No | No | Yes | No | No | No | No | 10(62.5) |
| Pulmonary effusion | No | Yes | Yes | Yes | No | No | No | No | Yes | No | No | No | Yes | Yes | Yes | Yes | 8(50) |
| Cord high density shadows | Yes | Yes | No | No | Yes | Yes | No | No | Yes | No | No | Yes | Yes | No | No | No | 7(43.75) |
| Pleural thickening | Yes | No | Yes | Yes | No | No | No | No | No | No | No | No | No | No | Yes | No | 4(25) |
| Atelectasis | No | Yes | No | Yes | No | No | No | No | No | No | Yes | No | No | No | No | Yes | 4(25) |
| Consolidation of lung | No | No | Yes | No | No | No | No | No | No | No | No | No | Yes | No | No | No | 2(12.5) |
| **Blood routine** |  |  |  |  |  |  |  |  |  |  |  |  |  |  |  |  | **Reference**  **range** |
| Hemoglobin (g/L) | decreased（91） | Nomal | decreased（98） | decreased（103） | decreased（101） | decreased（116） | decreased（83） | decreased（92） | decreased（78） | decreased（87） | decreased（55） | decreased（120） | decreased（76） | decreased（93） | decreased（86） | decreased（39） | 130-175 |
| White blood cell count (×10^9/L) | Nomal | Nomal | Nomal | decreased（2.95） | Nomal | Nomal | Nomal | decreased（2.74） | decreased（2.88） | Nomal | Nomal | Nomal | Nomal | Nomal | Nomal | Nomal | 3.5-9.5 |
| Lymphocyte count (×10^9/L) | decreased（0.94） | decreased（0.64） | decreased（0.76） | decreased（0.91） | decreased（0.11） | decreased（0.64） | decreased（0.49） | decreased（0.56） | decreased（0.3） | decreased（0.72） | decreased（1.09） | decreased（0.62） | decreased（0.33） | decreased（0.91） | decreased（0.65） | decreased（1.05） | 1.1-3.2 |
| **Coagulation function** |  |  |  |  |  |  |  |  |  |  |  |  |  |  |  |  |  |
| APTT (s) | Nomal | Nomal | Nomal | Nomal | decreased（19.2） |  | Nomal | Nomal |  | Nomal | Nomal | Nomal | increased（44.5） | increased（43.3） | increased（43.3） | increased（67.8） | 20-40 |
| Prothrombin  time (s) | Nomal | Nomal | Nomal | Nomal | Nomal |  | Nomal | Nomal |  | increased（13.1） | Nomal | Nomal | increased（15.4） | increased（16.7） | increased（13.7） | increased（19.3） | 9-13 |
| D-dimer (mg/L) | increased（0.80） | increased（4.20） | increased（0.80） | increased（0.91） | increased（3.11） | Not tested | increased（5.59） | Nomal | Not tested | increased（1.46） | increased（1.57） | increased（2.11） | increased（3.71） | increased（7.22） | increased（1.28） | increased（7.96） | 0-0.5 |
| **Blood biochemistry** |  |  |  |  |  |  |  |  |  |  |  |  |  |  |  |  |  |
| AST (U/L) | Nomal | Nomal | Nomal | Nomal | decreased（9） | Nomal | Nomal | decreased（14） | decreased（14） | Nomal | decreased（11） | Nomal | Nomal | increased（933） | Nomal | Nomal | 15-40 |
| ALT (U/L) | Nomal | Nomal | Nomal | Nomal | decreased（5） | Nomal | decreased（5） | Nomal | Nomal | Nomal | Nomal | Nomal | Nomal | increased（1782） | Nomal | Nomal | 9-50 |
| Albumin (g/L) | decreased（35.0） | decreased（37.80） | decreased（31.50） | decreased（29.40） | decreased（28.60） | decreased（33.60） | decreased（30.80） | decreased（34.40） | decreased（34.40） | decreased（33.40） | decreased（35.50） | decreased（34.30） | decreased（31.00） | decreased（35.70） | decreased（27.80） | decreased（21.40） | 40-55 |
| Serum  creatinine  (μmol/L) | increased（886.5） | increased（1319.1） | increased（987.9） | increased（1286.9） | increased（844.6） | increased（264.6） | increased（582.8） | increased（1047.6） | increased（1087.4） | increased（1416.6） | increased（1279.3） | increased（2194.1） | increased（2563.4） | increased（903.5） | increased（662.1） | increased（1845.5） | 41-73 |
| **Infection-related biomarkers** |  |  |  |  |  |  |  |  |  |  |  |  |  |  |  |  |  |
| C-reactive  protein (mg/L) | increased（35.00） | increased（20.00） | increased（51.90） | increased（43.6） | increased（27.9） | increased（54.00） | increased（22.5） | Nomal | increased（30.60） | increased（99.10） | increased（20.60） | increased（142.60） | increased（128.10） | increased（46.90） | increased（98.70） | increased（33.00） | 0-10 |
| Procalcitonin  (ng/mL) | increased（0.32） | increased（0.48） | increased（1.65） | increased（0.57） | increased（3.64） | increased（0.58） | increased（0.42） | increased（0.32） | increased（1.16） | increased（2.98） | increased（0.42） | increased（3.86） | increased（23.13） | increased（0.59） | increased（1.10） | increased（2.20） | 0-0.05 |
