# Supplementary material for: Characteristics and outcomes of hemodialysis patients with COVID-19: a retrospective single center study
Source: PeerJ. 2020 Nov 26;8:e10459. doi: 10.7717/peerj.10459 (PMC7700734; doi:10.7717/peerj.10459)
Supplement: Supplemental Information 2 [file peerj-08-10459-s002.docx]

**Table S2 Treatment of 16 hemodialysis patients with COVID-19**

| **Characteristics** | **Hemodialysis Patients with COVID-19 (n = 16)** | | | | | | | | | | | | | |  |  |  |
| --- | --- | --- | --- | --- | --- | --- | --- | --- | --- | --- | --- | --- | --- | --- | --- | --- | --- |
|  | **Patient 1** | **Patient 2** | **Patient 3** | **Patient 4** | **Patient 5** | **Patient 6** | **Patient 7** | **Patient 8** | **Patient 9** | **Patient 10** | **Patient 11** | **Patient 12** | **Patient 13** | **Patient 14** | **Patient 15** | **Patient 16** | **n (%)** |
| Oxygen  therapy | Yes | Yes | Yes | Yes | Yes | No | Yes | Yes | Yes | Yes | Yes | Yes | Yes | Yes | Yes | Yes | 15(93.75) |
| Antibiotic  treatment | Amoxicillin clavulanate potassium， Levofloxacin，Moxifloxacin | Levofloxacin | Moxifloxacin | Moxifloxacin,  Levofloxacin | Moxifloxacin, Levofloxacin | No | Levofloxacin | Cephalosporin, Moxifloxacin | No | Moxifloxacin, Levofloxacin | Moxifloxacin | Levofloxacin | Moxifloxacin, Ceftriaxone, Levofloxacin | Moxifloxacin, Levofloxacin | Cefoxitin, Levofloxacin, Cefoperazone sulbactam, Moxifloxacin | Moxifloxacin, Levofloxacin | 14(87.5) |
| Antiviral  treatment | Arbidol Tablets， Lopinavir and Ritonavir Tablets | Arbidol Tablets, Oseltamivir, Lopinavir and Ritonavir Tablets | Oseltamivir，Arbidol Tablets | Lopinavir and Ritonavir Tablets | Arbidol Tablets,Oseltamivir, Lopinavir and Ritonavir Tablets | No | Oseltamivir | Arbidol Tablets, Oseltamivi | Arbidol Tablets | Arbidol Tablets | Arbidol Tablets, Lopinavir and Ritonavir Tablets | Arbidol Tablets, Lopinavir and Ritonavir Tablets | Lopinavir and Ritonavir Tablets | No | Arbidol Tablets, Oseltamivir | Oseltamivir, Ribavirin | 14(87.5) |
| Traditional chinese medicine | Lianhua Qingwen Capsule | Toujiequwen Keli | Lianhua Qingwen Capsule | Lianhua Qingwen Capsule | Lianhua Qingwen Capsule | No | Lianhua Qingwen Capsule | Toujiequwen Keli | Lianhua Qingwen Capsule | Toujiequwen Keli | No | Lianhua Qingwen Capsule | Lianhua Qingwen Capsule | Lianhua Qingwen Capsule | Lianhua Qingwen Capsule | Lianhua Qingwen Capsule | 14(87.5) |
| Glucocorticoids | No | No | No | No | Methylprednisolone | No | No | No | Hydrocortisone | No |  | No | No | No | No | No | 2(12.5) |
| Intravenous  immunoglobulin  therapy | No | No | No | No | Yes | No | No | No | No | No | No | No | No | No | No | No | 1(6.25) |
