# Supplementary material for: Characteristics and outcomes of hemodialysis patients with COVID-19: a retrospective single center study
Source: PeerJ. 2020 Nov 26;8:e10459. doi: 10.7717/peerj.10459 (PMC7700734; doi:10.7717/peerj.10459)
Supplement: Supplemental Information 3 [file peerj-08-10459-s003.doc]

**Table 3 Treatment and clinical outcomes of hemodialysis and non-dialysis patients with COVID-19**

| **Characteristics** | **No. (%)** | | ***P* value** |
| --- | --- | --- | --- |
|  | **Hemodialysis Patients** | **Non-dialysis Patients** |  |
| Oxygen therapy | 15 (93.8) | 52 (83.9) | 0.542 |
| Mechanical ventilation | 2 (12.5) | 6 (9.7) | > 0.99 |
| Antibiotic treatment | 14 (87.5) | 47 (75.8) | 0.503 |
| Antiviral treatment | 14 (87.5) | 60 (96.8) | 0.388 |
| Traditional chinese medicine | 14 (87.5) | 56 (90.3) | 1 |
| Glucocorticoids | 2 (12.5) | 15 (24.2) | 0.503 |
| Intravenous immunoglobulin therapy | 1 (6.25) | 9 (14.5) | 0.644 |
| Length of stay (days) | 21 (15.5-30.1) | 14 (11-20) | 0.077 |
| Outcomes (death) | 3 (18758) | 5 (8.1) | 0.427 |

Data are reported as n (%) or median (IQR). The *P* value represents the difference between hemodialysis and non-dialysis patients. *P* value < 0.05 was considered significant difference.
